# Effects of UV Absorber on Zirconia Fabricated with Digital Light Processing Additive Manufacturing

**DOI:** 10.3390/ma15248726

**Published:** 2022-12-07

**Authors:** Jin-Ho Kang, Kumaresan Sakthiabirami, Hyun-Ah Kim, Seyed Aliakbar Hosseini Toopghara, Mee-Jin Jun, Hyun-Pil Lim, Chan Park, Kwi-Dug Yun, Sang-Won Park

**Affiliations:** 1 Department of Prosthodontics, School of Dentistry, Chonnam National University, Gwangju 61186, Republic of Korea; 2 Biomedical Evaluation and Research Centre, School of Dentistry, Chonnam National University, Gwangju 61186, Republic of Korea; 3 Department of Medical Engineering Joint Research, Chonnam National University, Gwangju 61186, Republic of Korea; 4 Department of Dental Hygiene, Gwangju Health University, Gwangju 62287, Republic of Korea

**Keywords:** UV absorbers, zirconia, additive manufacturing, cure depth, geometric overgrowth

## Abstract

This study evaluated the effect of UV absorbers on the dimensional accuracy of zirconia specimens fabricated by additive manufacturing using a digital light process. Zirconia suspension for additive manufacturing was prepared by setting the volume fractions (0, 0.005, 0.05, and 0.1%) of various UV absorbers. The effect of UV absorber content was evaluated through curing thickness, geometric overgrowth model design, linear deviation, and microstructure evaluation before and after sintering. Statistical analysis was performed by Kruskal–Wallis H and post-tested by the Bonferroni correction method. There was no significant difference in the cure depth according to the presence or absence of the UV absorber, the difference in geometric overgrowth was from 2.1 to 12.5%, and the overgrowth significantly decreased as the amount of added UV absorber increased. This result may contribute to improved precision of 3D multilayer ceramic products.

## 1. Introduction

With the development of digital dentistry, computer-aided design/computer-aided manufacturing (CAD/CAM) system zirconia prostheses have gained attention for satisfactory esthetics, high biocompatibility, and improved mechanical properties compared to existing dental ceramics [1].

Zirconia ceramic also has a variety of different applications other than dental implants and frameworks [2,3,4], such as electrolyte [5,6] and monolithic support for solid oxide fuel cells [7], as a part of cutting tools and blades [8], for elaborate molds [9] and other precision components in thermal and mechanical applications [10].

Currently, zirconia prostheses are fabricated by subtractive manufacturing using a CAD/CAM system. A completely sintered zirconia prosthesis is obtained by milling and sintering a pre-sintered zirconia block. Studies show that 15–30% (approximately 20%) of linear shrinkage occurs due to sintering [11]. However, the exact shrinkage rate is compensated for by setting the enlargement rate according to the manufacturer’s instructions for the zirconia block. Prosthesis fabrication through subtractive manufacturing has certain disadvantages, including the consumption of materials from milling burs or remaining blocks, the possibility of microcracking due to surface roughness or defects, and difficulty reproducing complex structures [12,13,14,15].

Three-dimensional printing techniques (or additive manufacturing) have gained popularity in various fields, such as temporary prostheses, splints, and model manufacturing, due to the ability to consume less material than subtractive manufacturing and reproduce complex structures [16,17,18]. With the recent development of additive manufacturing, studies have aimed to apply these techniques to prosthetic manufacturing in dentistry. Studies using various 3D printers, such as photopolymerization digital light processing (DLP), stereolithography apparatus (SLA), selective laser sintering (SLS), and spraying polyjet printers, are being introduced for zirconia additive manufacturing [19,20,21,22,23].

The photopolymerization method shows relatively higher precision, faster manufacturing speed, and higher surface roughness among these techniques. Therefore, it is more likely to be used in manufacturing dental prostheses [24]. The DLP uses a projector, modified to produce a specific wavelength, and a digital micromirror device to cure the liquid resin in layer units. Although the precision is lower than the SLA method, using a laser scanner, DLP shows excellent accuracy in small prints, such as crowns, and is advantageous due to its high manufacturing speed and relatively inexpensive equipment cost [7,25].

In dentistry, strength and precision are enormous challenges when manufacturing zirconia prostheses by DLP additive manufacturing. The process requires a zirconia photopolymer suspension, generally including zirconia powder, photosensitive resin, photoinitiator, and dispersant. For zirconia to have high strength, a high zirconia volume fraction is required, creating difficulty in obtaining an appropriate degree of dispersion and viscosity [26]. After mixing, it is crucial to have a well-dispersed suspension with relatively low viscosity to ensure precise additive manufacturing [27,28]. A study by Jang et al. [29] on the production of zirconia using DLP additive manufacturing showed that a volume fraction of zirconia of 58 vol% was the maximum range possible to achieve homogeneous mixing. As the volume fraction increases, the 3-point bending strength increases. However, the study reported that the viscosity increased rapidly to 56 vol%.

Geometrical overgrowth was introduced as a factor affecting manufacturing accuracy using DLP additive manufacturing zirconia [29,30]. Geometrical overgrowth refers to light scattering during the photopolymerization of the zirconia suspension, resulting in the over-curing of the surrounding. Light scattering occurs due to the high refractive index, polycrystalline grain structure, and relatively large grain size of zirconia [29,30,31]. A previous study [29,32] reported the evaluation of curing depth and geometrical overgrowth of additive manufacturing according to the volume fraction of zirconia and showed that the geometrical overgrowth by light scattering increased as the volume fraction increased, and a decreased curing time caused light scattering. A reduction in curing time could adversely affect cure depth. The photopolymerization method in ceramic additive manufacturing showed that overgrowth increased as the exposure time and area increased [30,33]. Based on the previous report, once the slurry and printing parameters were optimized, the layer lines played a minor role in the strength [34], which eventually highlights the importance of printing overgrowth optimization. Literature shows few laboratory studies on the geometrical overgrowth of additive manufacturing of zirconia. Mitteramskogler et al. [30,35] added a UV absorber to the suspension composition to suppress overgrowth due to light scattering during ceramic additive manufacturing. The UV absorber absorbs UV rays and converts them into thermal energy [36]. It can also control the penetration depth of UV rays and prevent proper dispersion and over-curing [37,38,39]. While the UV absorber is mainly studied as a polymer stabilizer by adding it to the photopolymer suspension, further studies on its effectiveness as a light scattering inhibitor and controlling overgrowth in zirconia additive manufacturing are needed.

Additive manufacturing using commercially available photopolymer resin for 3D printing not only controls but also compensates for the curing contraction of the resin and has shown clinically acceptable precision for manufacturing crowns and other prostheses [27]. It is necessary to compensate for the shrinkage due to sintering and evaluate and control geometrical overgrowth to obtain stability and precision in dental prostheses during the additive manufacturing of zirconia prostheses. Therefore, this study aimed to assess the degree of geometrical overgrowth based on the UV absorber of DLP additive manufacturing zirconia and also looks to increase the accuracy of additively manufactured zirconia prostheses in dental practice. Moreover, we aimed to assess the influence of UV absorbers on the dimensional accuracy of zirconia shaping fabricated by DLP additive manufacturing.

## 2. Materials and Methods

### 2.1. Zirconia Suspension Preparation

Zirconia photopolymer suspension was prepared based on the acrylate series commercialized with zirconia powder (TZ-3Y; Tosoh, Tokyo, Japan), and Table 1 shows the mechanochemical properties of each material [29]. The other additive agents included a photoinitiator (Irgacure 819; Ciba Specialty Chemicals, Basel, Switzerland) and dispersant (BYK-180; BYK Inc., Wesel, Germany) added based on the previous study [29]. The volume fraction of the zirconia was calculated to be 54 vol% to prepare a suspension. Trimethoxysilane (MTMS; Sigma-Aldrich Inc., Saint Louis, MO, USA) was added for silane treatment, and hydroxyphenyl-triazine (Tinuvin-477; BASF, Ludwigshafen, Germany) was added as a UV absorber (Orange 3, Sigma-Aldrich, Saint Louis, MO, USA) [40] Table 2 shows the composition of zirconia suspension for each group. The UV absorber volume fraction was divided into four groups, with added 0, 0.005, 0.05, and 0.1 vol%, respectively. A planetary centrifugal mixer (ARV-310; Thinky Corp., Tokyo, Japan) was used for homogenous mixing.

### 2.2. Fabrication of Specimen via Additive Manufacturing

**Figure 1:**
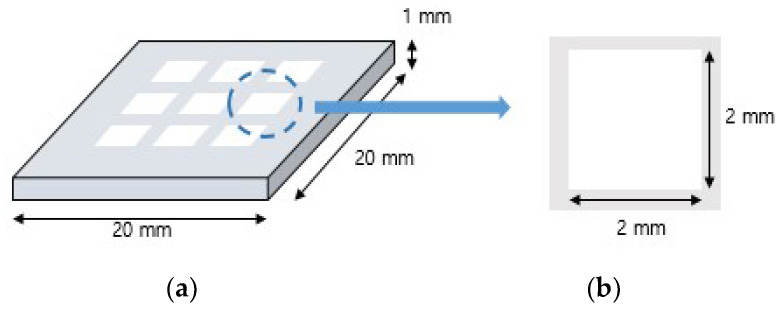
(**a**) Schematization of the STL file of the specimen. (**b**) The square hole (2 × 2 mm) for geometrical overgrowth measurement.

Figure 1 shows the specimen for this study was designed in nine hollow structures as a 2 mm square of 20 mm × 20 mm × 1 mm using CAD software (SolidWorks 2016, Dassault Systems Corp., Vélizy-Villacoublay, France) was then converted to a standard tessellation language (STL) file for additive manufacturing. Zirconia specimens were prepared using DLP equipment (Octave Light R1, Octave Light Ltd., Shatin, Hongkong) (wavelength 365–405 nm), setting the thickness of each layer to 50 µm and the exposure time to 4 s (*n* = 5) [31].

### 2.3. Geometrical Overgrowth Evaluation

**Figure 2:**
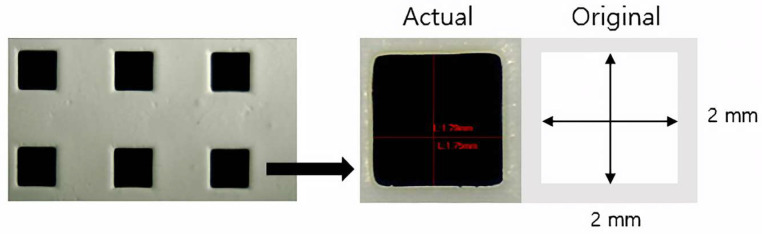
Measurement of linear deviation using a stereoscopic microscope.

Geometrical overgrowth occurs by curing a specimen into a wider area than the existing area exposed to the light source resulting from the light scattering effect of zirconia particles [31]. This experiment measured the degree of reduction of the nine hollow square structures. The overgrowth observed in the experiment was compared to the STL file standard in length and area. Figure 2 shows the length was measured using a stereomicroscope (EGVM-452M; EG Tech, Gwangyang, South Korea) by one person at a specific position throughout the study (*n* = 5).

**Figure 3:**
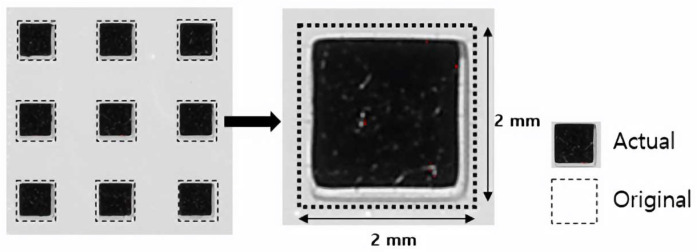
Measurement of area deviation using image analysis software.

Figure 3 shows the area measured using image analysis software (ImageJ software, National Institutes of Health, Bethesda, MD, USA). Geometrical overgrowth was quantified by calculating the difference between the actual measured and the designed size (2 mm).

### 2.4. Cure Depth

**Figure 4:**
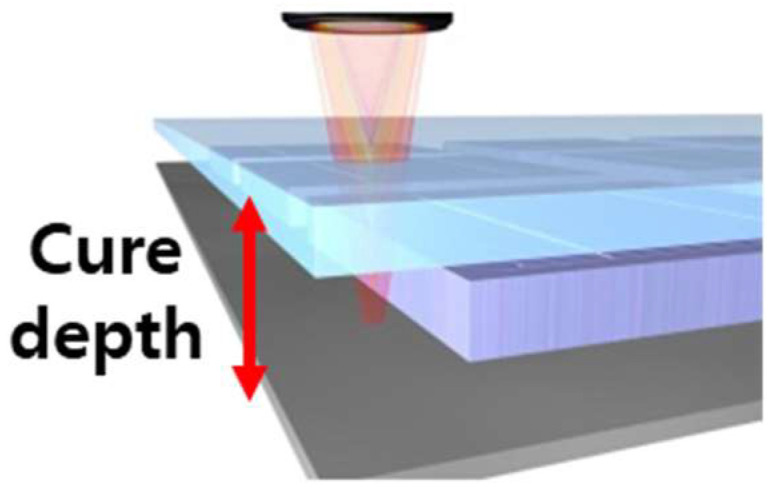
Schematic representation of curing depth of zirconia suspension by UV exposure.

Cure depth was measured to evaluate the effect of the UV absorber during lamination. Under the same conditions as the DLP 3D printer, a distance of 20 cm, a light of 100 mW/cm², and a light exposure time of 4 s were maintained. Figure 4 shows the final cured thickness (*n* = 3) based on the volume fraction of the UV absorber, which was measured using digital vernier calipers (*n* = 3).

### 2.5. Microstructural Analysis

The conditions for degreasing and sintering were set based on the results of previous experiments [39]. The microstructure and surface of the specimens were observed before and after sintering, and the defects were observed using a field emission scanning electron microscope (FE-SEM; JEOL) (ISO-13356) based on the UV absorber volume fraction [38].

### 2.6. Statistical Analysis

All statistical analyses were performed using SPSS 21.0 (SPSS Inc.; Chicago, IL, USA). All results were tested for significance at the *p* < 0.0083 level. As normality was not satisfied in the Shapiro–Wilk test, the Kruskal–Wallis, a nonparametric test, was performed. The post hoc test was performed using the Bonferroni correction method.

## 3. Result and Discussion

### 3.1. Geometrical Overgrowth Evaluation

It is essential to evaluate the strength and precision of additive manufacturing of zirconia dental prostheses using DLP. Geometric overgrowth, caused by over-curing resulting from the light scattering effect, is a factor that can affect precision. Light scattering is affected by the volume fraction of zirconia, the difference in refractive index between the zirconia powder and the medium, and the curing time [28,29,30]. Jang et al. [29] reported that zirconia has a refractive index of 2.1, which is 20–27% higher than silica and alumina. Thus, the degree of light scattering is large, and the cure depth is limited. In this study, a UV absorber was added to the 54 vol% zirconia photopolymer suspension to evaluate the degree of overgrowth. The cure depth was measured to ensure sufficient curing for additive manufacturing.

**Figure 5:**
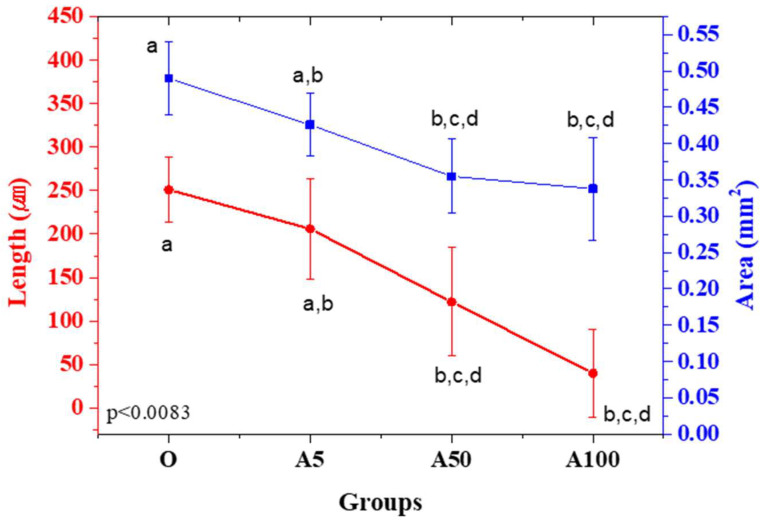
Comparison of length (μm) and area (mm^2^) of the prepared sample. Different letters indicate significant differences and when the same letters included there is no significant differences. (*p* < 0.0083).

**Figure 6:**
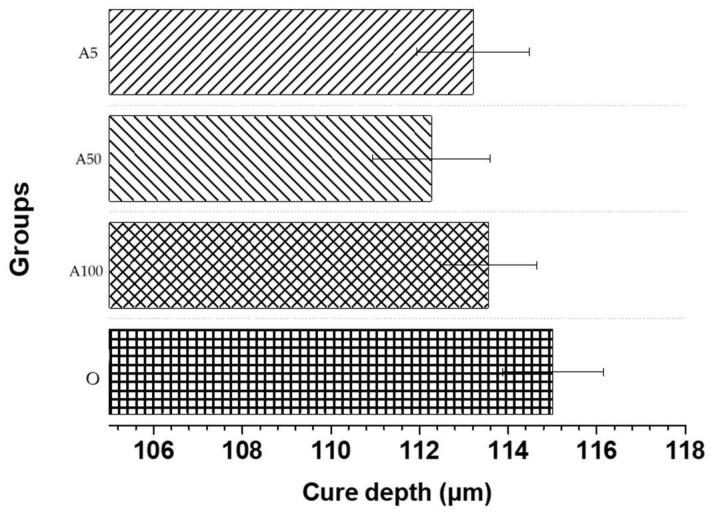
The bar graph represents the cure depth (μm) of the prepared sample.

Figure 5 and Figure 6 and Table 3 show the degree of overgrowth by subtracting the length and area of each specimen measured manufactured based on the UV absorber ratio by the designed value. In all the groups, the void structure decreased and showed an overgrowth pattern. As the UV absorber ratio increased, the degree of overgrowth tended to decline. In terms of length, the average degree of overgrowth was 40 μm in the A100 group and 251 μm in the O group. Regarding the ratio, the length showed an overgrowth rate of 2.1% in the A100 group and 12.5% in the O group, and the area showed an overgrowth rate of 8.5% in the A100 group and 12.25% in the O group. Significant differences were observed in the length and areas of the O group and the A50 and A100 groups without a UV absorber (*p* < 0.0083). No significant difference was observed based on the UV absorber ratio.

The results highlight that the group prepared by adding 0.05% and 0.1% UV absorber to the zirconia suspension (A50 group, A100 group) showed a significantly greater degree of overgrowth in length and area than the group without the addition of UV absorber (Group O). Our previous study [29] measured geometrical overgrowth according to the volume fraction of zirconia, showing an overgrowth rate of 33.52% at 54 vol%, higher than the results obtained in the current study. Despite the differences in the composition of the UV absorber and suspension, a geometrical overgrowth can be considered a factor that influences the larger exposure area and longer cure time than the specimen in this study. Geometrical overgrowth can be affected by the presence or absence of a UV absorber, as shown in this study, in addition to factors such as curing time, light quantity, exposure area, and difference in suspension composition [7,24,29,30].

The results show that the geometrical overgrowth was the lowest in the A100 group, containing 0.1% UV absorber, with overgrowth rates of 2.1% and 8.5% in length and area, respectively. When the overgrowth rate of the length was lower, the overgrowth was not constant by region, and the periphery of the rectangle tended to be rounded. Thus, we assumed that there was a difference in the area when the length was measured at a certain intermediate position. Additional studies are needed on the degree of overgrowth and the direction and pattern of overgrowth according to the specimen shape for precise additive manufacturing of zirconia.

### 3.2. Cure Depth

Zirconia prostheses are generally manufactured by setting the layer thickness between 25 and 100 μm during DLP additive processing [17]. The pre-sintered body is additively manufactured according to the fixed layer thickness. If the curing depth is lower than the layer thickness, delamination may occur between layers, causing micro-defects and deterioration of properties [7]. Therefore, attempts to suppress light scattering by adding a UV absorber or adjusting the curing time should consider whether the cure depth is sufficient beyond the set layer thickness.

In this study, the thickness of the laminated layer was set to 100 µm and measured to check whether adding a UV absorber affects layering. Figure 6 and Table 4 show there was no significant difference in the cure depth based on the UV absorber. All groups showed a cure depth of 100 μm or more. The cure depth was obtained at a uniform intensity of light and distance and measured using digital vernier calipers (*n* = 3). Surface defects and microstructures were analyzed using a field emission scanning electron microscope (FE-SEM; JEOL). The specimens were sintered at 1450 °C to observe the surface after sintering.

### 3.3. Microstructural Analysis

**Figure 7:**
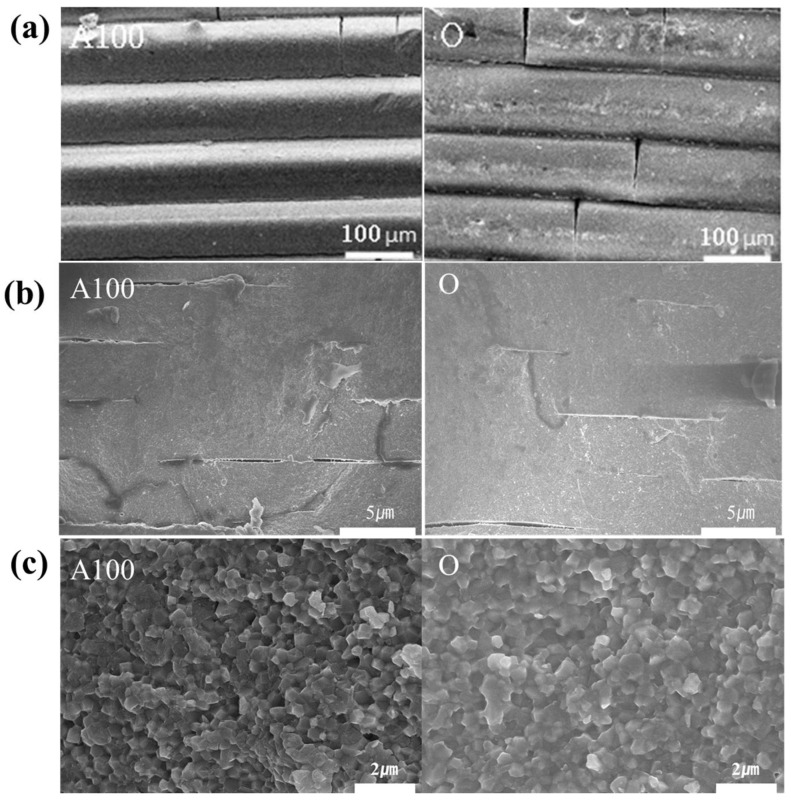
FE-SEM surface image at magnifications of (**a**) green bodies, (**b**) sintered specimens and (**c**) sintered specimens of high magnification.

Figure 7 shows the cross-sectional microstructure of the zirconia specimen before and after sintering between the final selected A100 group specimen and the control group O group specimen. It confirmed that a 100 µm layer and slight microcracks were visible before sintering in both groups (Figure 7a). It is judged that the microcracks are generated during the cleaning process after printing is completed, suggesting caution is needed even during the post-treatment process. In Figure 7b, the cross-section of each specimen after sintering was observed. After sintering, it was confirmed that each printing layer confirmed before sintering had healing and simultaneous defects. It is judged that defects occurred due to the characteristic of zirconia shrinking during heat treatment. Compared to the control group, the lower cure depths of A100 did not provide adhesion to cause perfect healing between each layer. Figure 7c shows the high magnification of each sintered specimen. The grain sizes of 0.46 (±0.06) and 0.58 (±0.083) nm of the A100 group and O group can be confirmed, and also, it is confirmed that group O shows a difference of about 100 nm. However, there was no significant difference in each group. It is due to similar grain growth and powder characteristics since the experiment was performed under the same degreasing and sintering conditions.

## 4. Conclusions

This study investigated the degree of geometrical overgrowth when a UV absorber was added to the additive manufacturing of zirconia using DLP. When evaluating the overgrowth rate, the length measurement ranged from 2.1% to 12.5%, and the area measurement ranged from 8.5% to 12.25%. Results showed a significant difference in the overgrowth between the groups without the UV absorber (Group O) and the group with 0.05% and 0.1% (A50 group and A100 group). The degree of overgrowth was the smallest in the A100 group and the highest in the O group. Length measurement revealed an average value of 40 μm in the A100 group and 251 μm in the O group. The average area was 0.490 mm^2^ in group O. There was no significant difference in the cure depth in the groups with and without the UV absorber. Cure depths of 100 μm or more were observed in all groups. No surface defects or laminated zirconia layers were observed in the specimen after sintering in all groups. Thus, the geometrical overgrowth of pre-sintered bodies decreased when the UV absorber was added to the DLP-based additive manufacturing of zirconia, suggesting the possibility of increased accuracy of additive manufacturing of zirconia. Thus, sintering shrinkage compensation, reflecting geometrical overgrowth, is necessary to increase the accuracy in the additive manufacturing of zirconia.

## Figures and Tables

**Table 1:** Materials and their properties.

	Materials	Density (g/mL)	Refractive Index (325 nm)	Viscosity (25 °C, mPas)
Ceramic	Zirconia	6.05	2.15	-
Photopolymer	HDDA *	1.02	1.45	9
	IBA **	0.98	1.47	8
	PBPGDA ***	1	1.44	15
Additive agents	Photoinitiator	1.19–1.21	-	-
	Dispersant	1.075	-	-
Silane coupling agent	MTMS ****	0.955	1.371	-
UV absorber	Hydroxyphenyl-triazine	1.08	-	-

* 1,6-Hexanediol diacrylate, ** Isobomyl aceylate, *** Propoxylated neopentyl glycol diacrylatemtms, and **** Methyltrimethoxysilane.

**Table 2:** Composition of zirconia suspension.

Group	Zirconia	Photopolymer	Silane Coupling Agent	Dispersant	UV Absorber	Total (Vol%)
o	54	27.62	6.28	12.1	0	100
A5		27.60			0.005	100
A50		27.43			0.05	100
A100		27.24			0.1	100

**Table 3:** The mean and standard deviation of length and area.

Group	Length (μm)	Area (mm^2^)
A5	20 ± 657.4	0.426 ± 0.043
A50	122 ± 62.3	0.355 ± 0.051
A100	40 ± 50.4	0.338 ± 0.071
O	251 ± 37.1	0.490 ± 0.050

**Table 4:** The mean and standard deviation of cure depth.

Group	Cure Depth (μm)
A5	113.55 ± 1.09
A50	112.27 ± 1.33
A100	113.21 ± 1.27
O	115.01 ± 1.14

## Data Availability

All the data have been illustrated in the manuscript.
